# Effect of icariin on ovarian cancer: a combined network pharmacology and meta-analysis of *in vitro* studies approach

**DOI:** 10.3389/fphar.2024.1418111

**Published:** 2024-12-20

**Authors:** Shang-Mei Cao, Bo-Lin Chen, Zhen-Zhen Zou, Shao-Zhe Yang, Xiu-Hong Fu

**Affiliations:** Luohe Central Hospital, The First Affiliated Hospital of Luohe Medical College, Henan Key Laboratory of Fertility Protection and Aristogenesis, Shaoling District, Luohe, China

**Keywords:** anti-tumour, ovarian cancer, network pharmacology, meta-analysis, icariin

## Abstract

**Introduction:**

An abundance of experimental evidence indicates that icariin (ICA) could potentially exert an anti-tumor effect on ovarian cancer (OC). Nevertheless, the reliability of this evidence remains ambiguous. This study aimed to explore the impact of ICA on OC and the underlying mechanisms.

**Methods:**

Bioinformatics analysis was employed to pinpoint ICA-targeted genes and signaling pathways implicated in OC, utilizing network pharmacology. Subsequently, PubMed, EMBASE, and Web of Science databases were systematically searched from 2001 through June 2023 for *in vitro* trials evaluating the anti-tumor efficacy of conventional ICA versus placebo in OC. The pathways and genes identified in the literature were recorded, and the therapeutic targets were statistically analyzed and compared with the predicted targets from network pharmacology to confirm the precision of the targets.

**Results and Discussion:**

Fourteen target genes were validated with success. The pathways corresponding to the remaining genes—excluding these 14—were analyzed and found to be primarily associated with cell apoptosis, anti-tumor, and other related pathways. Out of the 76 studies retrieved, eight fulfilled the inclusion criteria. The subsequent meta-analysis suggested that ICA treatment was significantly correlated with reduced cell growth and induced apoptosis. This study demonstrated a certain efficacy of ICA compared to placebo in enhancing anti-tumor outcomes, characterized by increased abilities in reducing cell growth and inducing apoptosis. The pathways involved in the therapeutic effect may be linked to cell apoptosis and anti-tumor mechanisms.

## 1 Introduction

Ovarian cancer (OC) ranks as the third most prevalent form of gynecological tumor (Zheng et al., 2021). The majority of OC cases arise from epithelial cells. Research has indicated that exposure to estrogen and progesterone may elevate the risk of OC, particularly among postmenopausal women (Zhang Y. F. et al., 2022). This malignancy can lead to reduced fertility in women (Zhang et al., 2021). In clinical settings, numerous medications that stimulate follicular development are hormone-based (Zhang et al., 2024), However, for individuals with OC, drugs that neither encourage cancer cell proliferation nor follicular development are preferable for those seeking to maintain fertility while managing the disease (Yu et al., 2023). Consequently, identifying such medications is a pressing necessity.

Over recent years, the advancement of clinical research and the comprehensive adoption of standardized diagnostic and treatment protocols have led to the widespread adoption of gene detection technologies, including BRCA and HRD (Morand et al., 2021). Moreover, the utilization of targeted therapeutic agents has ushered OC into an era of precision medicine. The full-course management treatment model, which encompasses surgery, chemotherapy, and maintenance therapy, has also seen continuous progress, offering increasing benefits to a growing number of patients and their families (Munoz-Zuluaga et al., 2020). In the realm of pharmaceuticals, ongoing research is being conducted, with medical researchers dedicated to discovering improved drug treatment options for patients (Moubarak et al., 2022).

Protecting the fertility in cancer patients has become an increasingly important issue. It is particularly difficult to protect fertility without stimulating cancer cell proliferation. Traditional Chinese medicine has played a unique role in this regard, becoming the first choice for many cross-disease patients due to its wide range of target areas and bidirectional regulatory function (Lu et al., 2023). The relatively minor side effects, high-quality and affordable nature of traditional Chinese medicine promote its clinical use. Many effective traditional Chinese medicines have been discovered. The discovery of Chinese medicines has been made. Further development of traditional Chinese medicines or herbal extracts would be an effective strategy for optimizing treatment plans for ovarian cancer patients seeking fertility (Chelliah and van der Graaf, 2022).

Although traditional chemotherapy drugs have strong anti-cancer effects, they also have significant side effects. Many patients cannot tolerate these side effects and often give up treatment after a few cycles of chemotherapy, choosing targeted drug therapy instead. Although some targeted drugs are effective and have fewer side effects compared to traditional chemotherapy drugs, they are more expensive and have a single target; thus, they cannot have a common effect on other diseases, such as monoclonal antibody drugs like Olaparib (Gao et al., 2022). Traditional Chinese classical prescriptions for the treatment of OC have multiple targets and relatively few side effects but their components are complex and difficult to separate and extract, such as Guizi-Fulling Wan (Wang X. et al., 2022). Extracts from Chinese medicine have significant therapeutic effects but many side effects, such as Paclitaxel (Lau et al., 2020), Bufalin (Dou et al., 2021), etc. Some drugs are still in the research stage, including synthetic drugs and traditional Chinese medicine extracts, which only have anti-tumour effects. These include Agrimonolide (Liu et al., 2022), Momordica charantia (Chan et al., 2020), Curcumin (Sun and Fang, 2021), FBP1 (Li et al., 2021), etc., and these do not have a strong promotional effect on female fertility. However, ICA not only has anti-tumour effects but also has protective effects on fertility via improving oocyte quality (Szabó et al., 2022). Therefore, ICA is the best choice for OC patients who are seeking fertility.

An extensive literature search revealed that the traditional Chinese medicine extract icariin (ICA) promotes the proliferation of ovarian granulosa cells and inhibits the proliferation of OC cells, and has a bidirectional regulatory effect on both normal and OC cells (Wang et al., 2019). ICA is the main effective component of the natural drug *Epimedii herba*, has a well-defined chemical structure, which has an active therapeutic effect on endocrine disorders, infertility and other reproductive diseases (Niu et al., 2022). ICA has a clear positive effect on the nervous system (Lifeng et al., 2023), reproductive system (Tao et al., 2017), circulatory system (Xie et al., 2015), digestive system (Zhang et al., 2008), respiratory system (Li et al., 2023), motor system (Liu and Fang, 2022), urinary system (Chen et al., 2022), endocrine and metabolic diseases (Su et al., 2023) and reproductive system diseases. It can also improve the quality of oocytes (Ye et al., 2017), has a clear anti-inflammatory (Jiao et al., 2018) and anti-oxidant (Park et al., 2022) effect, and can regulate endocrine metabolism (Wang et al., 2017), immune function (Zhao et al., 2019) and exert an anti-tumour effect (Xie et al., 2019).

However, the effect of this ICA on OC cells is still in the cell research stage and there is limited literature data to support its efficacy. Therefore, to accurately evaluate its effect on OC cells, a systematic evaluation of eight key literature reports was undertaken to analyse the pathways identified in the literature, gain a deep understanding of the molecular mechanism of ICA on OC and ascertain future research directions.

## 2 Materials and methods

We employed network pharmacology to pinpoint the efficacious compounds in *Epimedium*, and subsequently, through a review of the literature, we ascertained the principal components. We then performed a cross-analysis with ovarian cancer-related pathways to identify those pertinent to both. The validation of these pathways was executed through an examination of published literature, utilizing meta-analysis to ascertain the studies with the highest reference value. The detailed methodology is as follows:

In this investigation, bioinformatics was employed to pinpoint genes and signaling pathways targeted by ICA in the context of OC. Through gene enrichment analysis, the study identified the KEGG pathways associated with both ICA-targeted genes and OC, along with the hub genes (Zhou et al., 2022). Employing a simulated approach, this research innovatively applied network pharmacology to forecast the therapeutic targets of ICA for OC treatment. Subsequently, a systematic evaluation of existing animal studies on ICA’s efficacy in treating OC was conducted. The therapeutic targets reported in the literature were statistically analyzed and compared with the predicted targets from network pharmacology to validate the latter’s precision. A common gene was identified between the two sets of targets, confirming the accuracy of the network pharmacology predictions.

### 2.1 Compound database building

The Chinese Medicine System Pharmacology (TCMSP) database was used to identify the main active compounds of *Epimedii herba* (Zhong et al., 2022), including ICA. The parameters used to select the active ingredients were an oral bioavailability (OB) of 30% and a drug-likeness (DL) of 0.18. PubChem (https://pubchem.ncbi.nlm.nih.gov) was used to extract the chemical structure of each active compound using SMILES. The predicted targets were found using the SMILES string in SwissTargetPrediction (www.swisstargetprediction.ch) (Zhao Y. et al., 2022). A probability of 0.1 was the screening condition for the target protein to predict the components (Zhao M. et al., 2023). The targets acquired were standardized to gene names by querying the UniProt Database (http://www.uniprot.org) using the “*Homo sapiens*” species as a filter (Zhao H. et al., 2023).

### 2.2 Acquisition of potential OC targets

The DisGeNET (www.disgenet.org) and GeneCards (www.genecards.org) databases were used to identify the genes and mutation sites associated with human diseases (Zhang Y. et al., 2022). The Venn Diagram Tool v2.1.0 from the Bioinformatics platform (www.bioinformatics.com.cn) was used to identify the shared targets between the composition and the target (Zhang Q. et al., 2022).

### 2.3 Constructing a protein–protein interaction (PPI) network

The STRING (https://string-db.org/) database and Cytoscape v3.7.2 software were used to construct the PPI network (Zhang et al., 2023).

### 2.4 Gene Ontology (GO) annotation and Kyoto Encylopaedia of Genes and Genomes (KEGG) enrichment analysis

The Database for Annotation, Visualization and Integrated Discovery (DAVID) was used for comprehensive gene annotation and resource analysis (https://david.ncifcrf.gov/home.jsp) (Zhang D. Y. et al., 2022). The preanalysis species was set to “*Homo sapiens*”. The Gene Ontology (GO) analysis encompassed three principal categories: biological processes (BP), cellular components (CC), and molecular functions (MF). Subsequently, the Kyoto Encyclopedia of Genes and Genomes (KEGG) pathway enrichment analysis was conducted. The enriched GO terms and the enrichment dot plots were derived from data analysis executed on the Bioinformatics online platform (Zeng et al., 2023).

### 2.5 Ingredient–target–pathway network construction

The ingredient-target-pathway network was constructed using Cytoscape software, incorporating intersection targets and pathways, and selecting six active ingredients. In this network, nodes of various colors signify different clusters, while the edges depict the interrelationships between these nodes (Yue et al., 2022).

### 2.6 Meta-analyses

The meta-analysis was conducted utilizing Review Manager 5.3, a tool provided by the Cochrane Collaboration, and adhered to the guidelines set forth in the Cochrane Handbook for Systematic Reviews of Interventions.

### 2.7 Article selection

The PubMed, EMBASE and Web of Science databases were searched. Only English-language articles published from 2001 to June 2023 were included. The databases were searched using the following search terms in titles and abstracts (also in combination with MESH terms): (“Icariin” OR “ICA”) AND (“cancer” OR “ovarian cancer” OR “cancer cell” OR “tumour” OR “malignancy” OR “cancer cell line” OR “neoplasm cell line”) AND (“apoptosis” OR “cancer apoptosis” OR “apoptotic cancer cell” OR “apoptosis *in vitro*” OR “caspase-3”). The electronic search was complemented by manual searching of the references in the included publications. A study was included if it met specific inclusion and exclusion criteria (Table 1). No restrictions in terms of the year of publication were applied.

**TABLE 1:** Inclusion and exclusion criteria.

Inclusion criteria	Exclusion criteria
1. All ovarian cancer cells model	1. Animals with co-morbidity, clinical trials, and *in vivo* models
2. ICA with all dosage and duration	2. ICA without batch number, other preparation of ICA
3. Cell growth reduction and apoptosis induction ability were the primary outcomes, the pathway involved were the secondary outcomes	3. Case studies, cross over studies, and studies without a separate Control group
4. Language: English	4. Not an original full research paper; duplicate publication; studies without full text

### 2.8 Study selection and data extraction

From the pool of eligible publications, the subsequent data—encompassing the first author, publication year, cell species, intervention duration and dosage, and outcome measures (including cell growth reduction capability, apoptosis induction capability, and pathways involved in OC)—were meticulously extracted by two independent authors (CB and SC). Discrepancies were reconciled through discussion, culminating in a consensus on all extracted data. Any disagreements among reviewers regarding data extraction were resolved via consultation with a third reviewer.

### 2.9 Statistical analysis

All the outcome measures were continuous data (e.g., cell growth reduction ability and apoptosis induction ability), so random-effects meta-analyses of these data with standardised mean differences (SMD) and 95% confidence intervals (95% CI) were performed. A *p-*value of *< 0.05* was considered statistically significant. The analysis method can be found in previous articles (Cao et al., 2022). Sensitivity analysis was conducted to evaluate whether a single study affected the overall effect size by removing one study at each stage. This was conducted when an experimental study had a Stata value of greater than 5. Additionally, publication bias was evaluated quantitatively using Begg’s and Egger’s tests, which were conducted using Stata. A significant publication bias was indicated if the *p*-value was *<0.05*. Review Manager 5.3 (the Cochrane Collaboration) and STATA 16.0 (Stata Corporation) were used for the analysis.

## 3 Results

### 3.1 Acquisition of potential OC targets by network pharmacology analyses

OC-related targets were identified using DisGeNET and GeneCards. Genes with a relevance score above 1 were selected in the Genecard database and genes with an nSNPs number above 1 were selected in the DisGeNET database. After removing duplicates, 1,389 genes were obtained.

### 3.2 Acquisition of ICA-targeted genes

**FIGURE 1:**
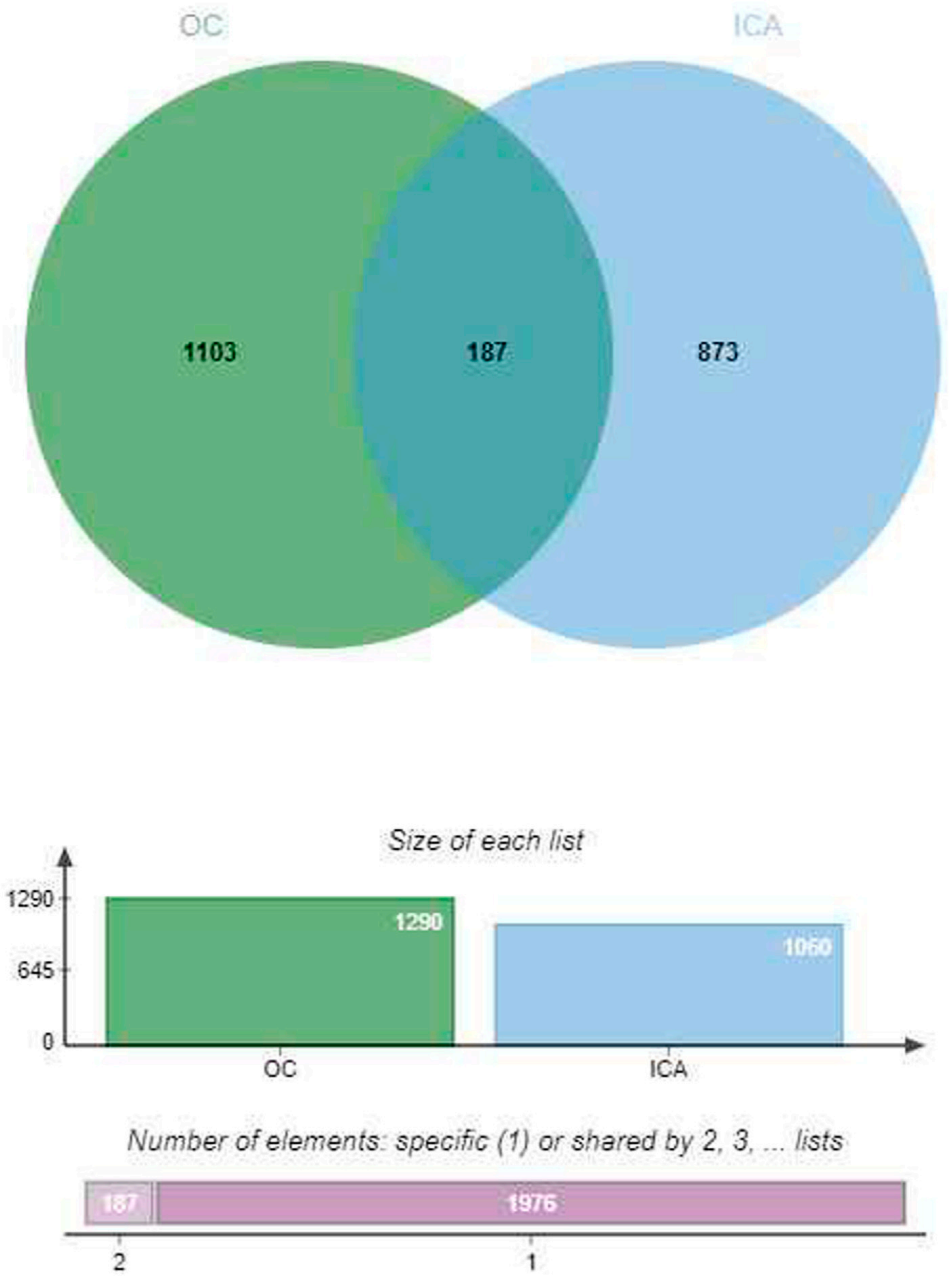
The Venn diagram. The intersection genes of ICA and OC.

Based on the findings from the TCMSP, the UniProt, and the SwissTargetPrediction database, 1,075 targets associated with ICA and 187 targets related to OC therapy were identified. Figure 1 illustrates the Venn diagram representing these two sets of targets, with the overlapping targets considered to encompass potential OC treatment targets.

### 3.3 Protein-protein interaction (PPI) network analysis and interaction networks

**FIGURE 2:**
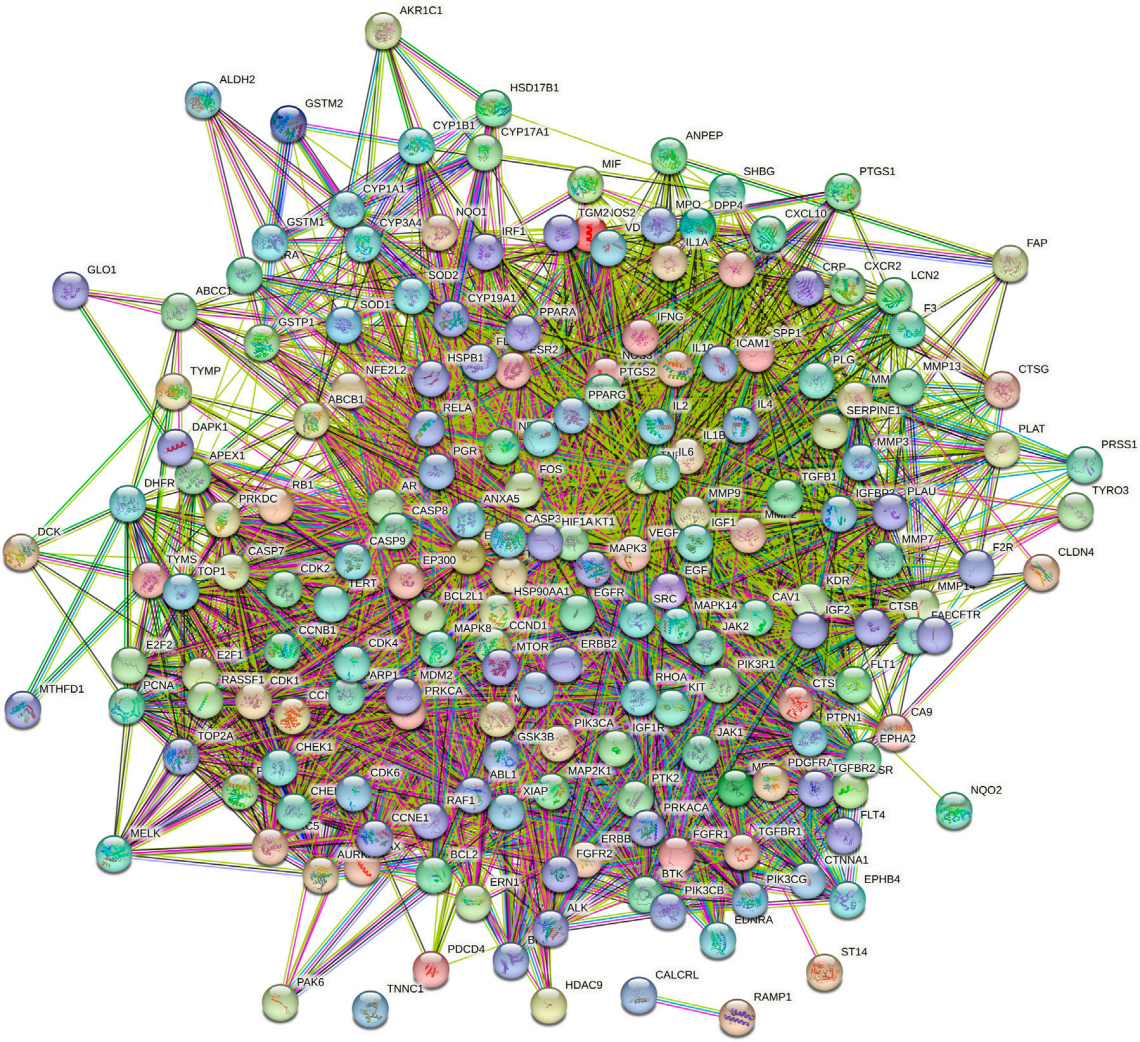
PPI Interaction network.

**FIGURE 3:**
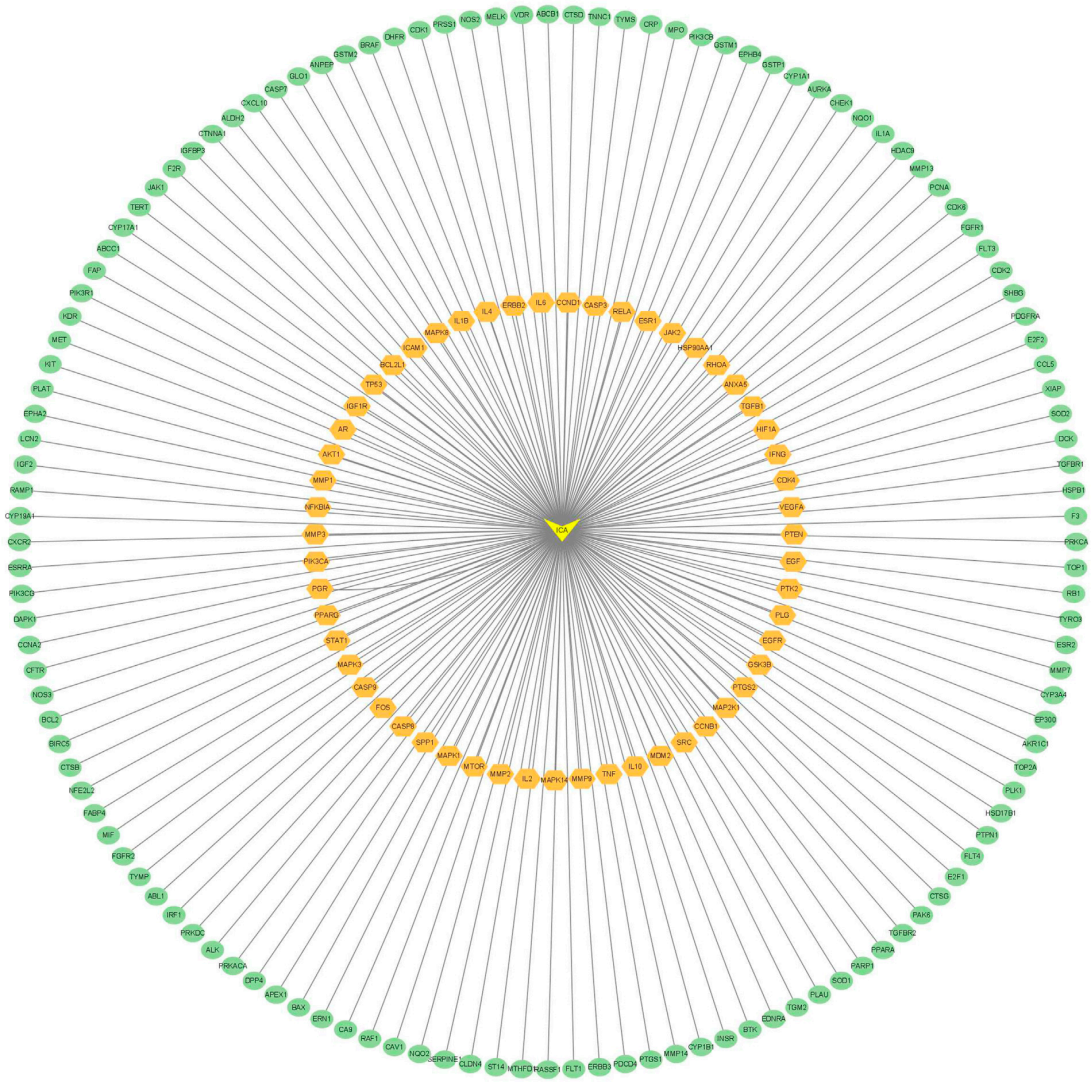
Construction of the ingredient-target-pathway network.

The intersection targets were imported into the STRING database to generate a PPI functional network. The PPI map contained 187 intersection targets (refer to Figure 2). Targets that were not connected to the network were subsequently removed, and the interactions of drug-targeted genes were constructed using Cytoscape version 3.7.2 (refer to Figure 3). Hub gene values were evaluated based on degree, betweenness, and closeness centrality. Genes colored yellow represented hub genes, which were most closely related to ICA, whereas genes colored green were ordinary genes, indicating secondary relevance to ICA. The roles of these targets in various diseases are discussed in detail below.

### 3.4 Gene Ontology (GO) analysis

Employing the Database for Annotation, Visualization and Integrated Discovery (DAVID), a metascape enrichment analysis was conducted on the intersecting genes to explore biological processes (BP), cellular components (CC), and molecular functions (MF) under Gene Ontology (GO) terms for ICA (refer to Supplementary Figures S1-S3). The *p*-value established the outcomes of the correlation test. The leading 20 terms within each category were organized by their logP values, ranging from smallest to largest, and a histogram was subsequently generated. The prominent terms identified included response to ethanol, positive regulation of smooth muscle cell proliferation, response to L-ascorbic acid, and response to hypoxia within BPs; extracellular region, extracellular exosome, mitochondrion, and extracellular space within CCs; and haem binding, identical protein binding, and insulin receptor substrate binding within MFs.

### 3.5 Kyoto Encyclopedia of Genes and Genomes (KEGG) pathway analysis

**FIGURE 4:**
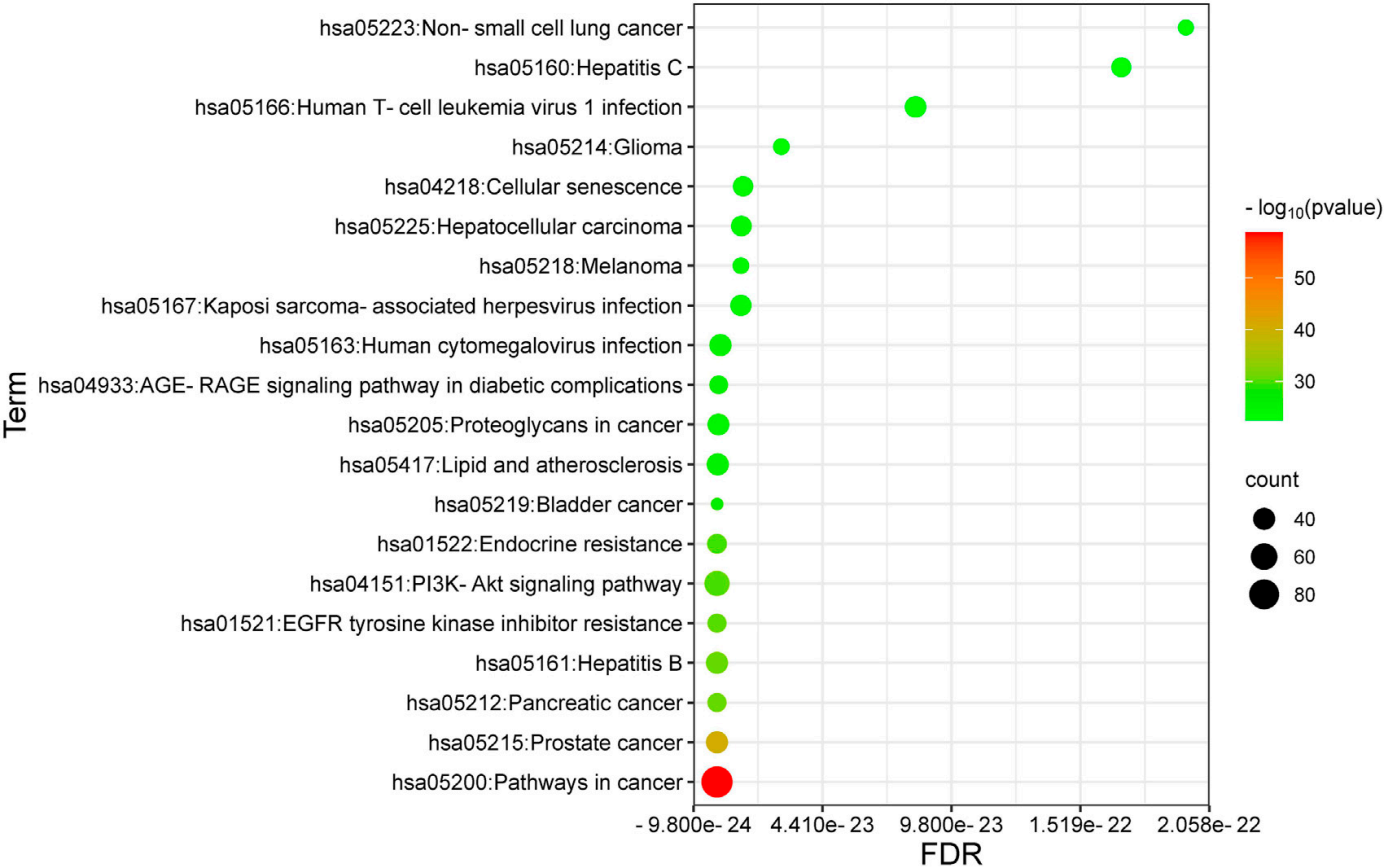
KEGG bubble chart. The top 20 KEGG category for ICA and OC intersection genes.

The top 20 shared KEGG pathways associated with ICA-targeted genes and osteosarcoma (OS) were identified using a bubble chart (Figure 4) and categorized based on the logP value, ranging from the smallest to the largest. The concentrations and *p*-values denote the highly correlated manner in which the active ingredients manifest their therapeutic effects on OS.

### 3.6 The pathways and molecules mentioned in the published articles

The mechanisms and pathways through which ICA influences OC were elucidated in eight articles. The genes coding for the proteins were pinpointed using the STRING database and subsequently contrasted with the genes derived from network pharmacology analysis conducted in the preceding phase. A significant overlap was observed between the two gene sets, with 14 genes that were both implicated in the literature and anticipated by network pharmacology: BIRC5, MMP9, CDK2, PIK3CA, ERBB2, MTOR, IL2, KDR, PTGS2, AKT1, TNF, PTEN, BCL2, and RELA (refer to Supplementary Figure S4). The pathways associated with the remaining genes from the 187 intersection targets—excluding these 14 genes—were scrutinized using the DAVID database, as depicted in Supplementary Tables S1–S3 and Supplementary Figure S5. The top 20 shared KEGG pathways corresponding to ICA and OC intersection genes that had not been previously validated were identified using a bubble chart (Supplementary Figure S6) and ranked based on their logP values from smallest to largest.

### 3.7 Study inclusion by meta-analysis

**FIGURE 5:**
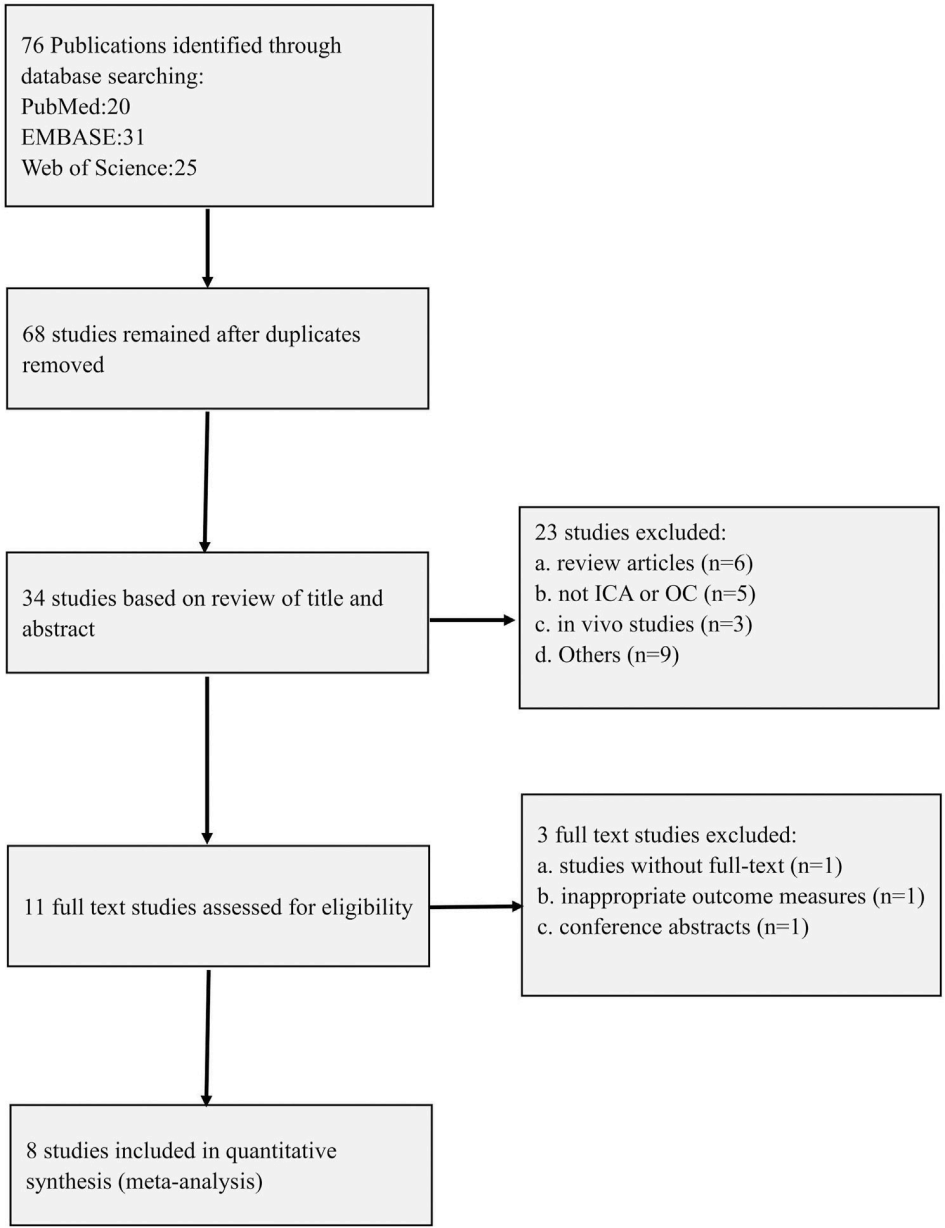
Flow diagram of the study selection process for this review.

The process of selecting studies is illustrated in Figure 5. An electronic search along with the examination of supplementary sources resulted in the identification of 76 publications. After removing duplicates, 68 studies were kept. Upon reviewing the titles and abstracts, 34 studies were excluded. As a result, eight full-text articles were considered suitable for assessment.

### 3.8 Study characteristics

The eight chosen *in vitro* studies were published between 2001 and 2023. The cell species encompassed SKOV3, SKVCR, A2780, and OVCAR3. The concentration of ICA varied from 6.5 µM to 80 µg·mL–1, and the duration of drug exposure ranged from 24 to 72 h. The primary characteristics and outcomes of the included studies are summarized in Table 2.

**TABLE 2:** Characteristics of the included studies.

Study year	N (runs)	Cell species	Concentration of ICA	Duration (h)	Method of apoptosis measure	Pathways
Wang 2020	3	SKOV3	100 µM	48	Annexin V/PI Flow cytometry	TNF, MMP9, STAT3, PIK3CA, ERBB2, MTOR, IL2,PTGS2, KDR, F2
Fu 2022	3	SKOV3	50 µM	48	Annexin V/PI Flow cytometry	miR-1-3p, TNKS2, Wnt, b-catenin, cyclinD1, Survivin
JIANG 2019	4	SKVCR	20 μg/mL	24	Annexin V/PI Flow cytometry	LC3B, Beclin-1, ATG5, p62
Jiang 2018	3	SKVCR	80 μg/mL	72	Western blot (caspase3)	Beclin-1, LC3I/LC3II, ATG3, AMBRA1, mTOR.
Li 2015	3	A2780	50 µM	48	Annexin V/PI Flow cytometry	PTEN, RECK, Bcl-2, miR-21
Fahmy 2020	4	SKOV3	10 µM	24	Annexin V/PI Flow cytometry	TNF-a
Alhakamy 2020	3	OVCAR3	6.5 µM	24	Annexin V/PI Flow cytometry	
GAO 2022	3	SKOV3	50 µM	48	Western blot (caspase3)	AKT, NF-κB, p65

### 3.9 Effect of ICA on proliferation inhibition ability

The publications assessed the capacity to diminish cancer cell proliferation using two distinct metrics. The first metric was the rate of OC cell growth reduction, while the second was the IC_50_ (μM) value for the OC cells. Due to the differing calculation methods and units of these two metrics, they were analyzed independently. The IC_50_ for the ICA-treated group compared to the control group was as follows: *n = 7, SMD =* −*8.28, 95% CI *(−*13.33,* −*3.23*)*, p = 0.001*; heterogeneity: *X* ^*2*^ *= 1.33, p = 0.25; I* ^*2*^ *= 25%*. The proliferation inhibition rate for the ICA-treated group compared to the control group was as follows: *n = 13, SMD =* −*7.56, 95% CI *(−*11.06,* −*4.06*)*, p < 0.0001*; heterogeneity: *X* ^*2*^ *= 1.52, p = 0.68; I* ^*2*^ *= 0%*.

**FIGURE 6:**
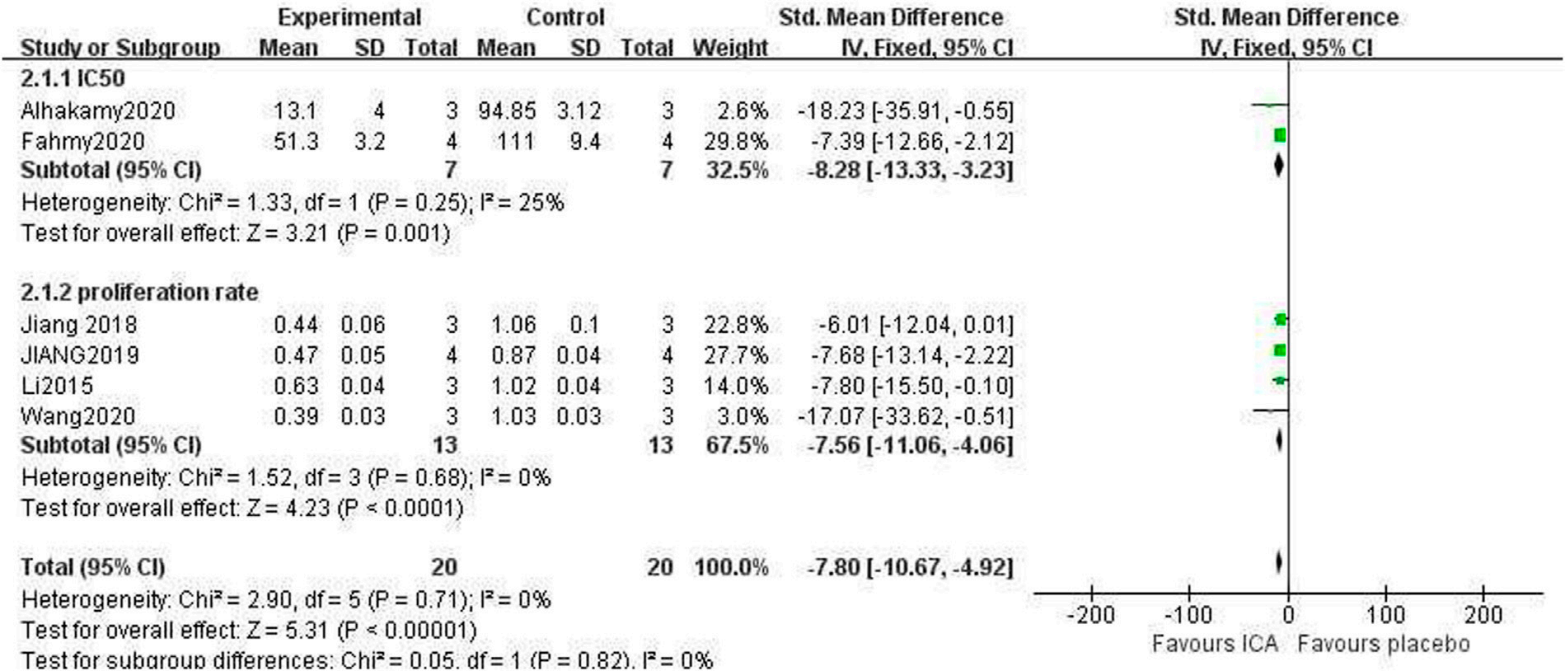
Pooled estimate of cell growth reduction ability in OC cells with ICA.

The test for subgroup differences result was *P = 0.82*, which was greater than the 0.05 threshold. The combined results indicated that ICA could significantly suppress cancer cell growth compared with the control group [*n = 20, SMD = −7.80, 95% CI *(−*10.67,* −*4.92*)*, p < 0.00001;* heterogeneity: *X* ^*2*^ *= 2.90, p = 0.71; I* ^*2*^ *= 0%*, Figure 6]. Funnel plots were constructed to show the asymmetry of the effects of ICA on cell growth reduction (Figure 8). Begg’s test revealed no statistical significance (*p = 0.060*), whereas Egger’s test revealed statistical significance [*95% CI *(−*2.62,* −*0.72*)*; p = 0.008*].

### 3.10 Effect of ICA on apoptosis promotion ability

The capacity to trigger apoptosis in cancer cells was assessed using two distinct methodologies as detailed in the publications. The first method involved the use of Annexin V/PI Flow Cytometry (FCM), while the second method utilized Western blot analysis to detect caspase-3 protein levels. Given that the calculation methods and units of measurement for these two techniques are different, two subgroups were established based on the varying units. The results for the apoptosis rate in the ICA-treated group compared to the control group, as determined by FCM, the results were as follows: *n = 17, SMD = 5.10, 95% CI *(*3.02, 7.19*)*, p < 0.00001;* heterogeneity: *X* ^*2*^ *= 2.54, p = 0.64; I* ^*2*^ *= 0%*. For the Western blot of caspase3 protein in the ICA-treated group compared to that in the control group, the results were as follows: *n = 6, SMD = −2.56, 95% CI *(*−4.43, −0.69*)*, p < 0.00001;* heterogeneity: *X* ^*2*^ *= 0.15, p = 0.70; I* ^*2*^ *= 0%*.

**FIGURE 7:**
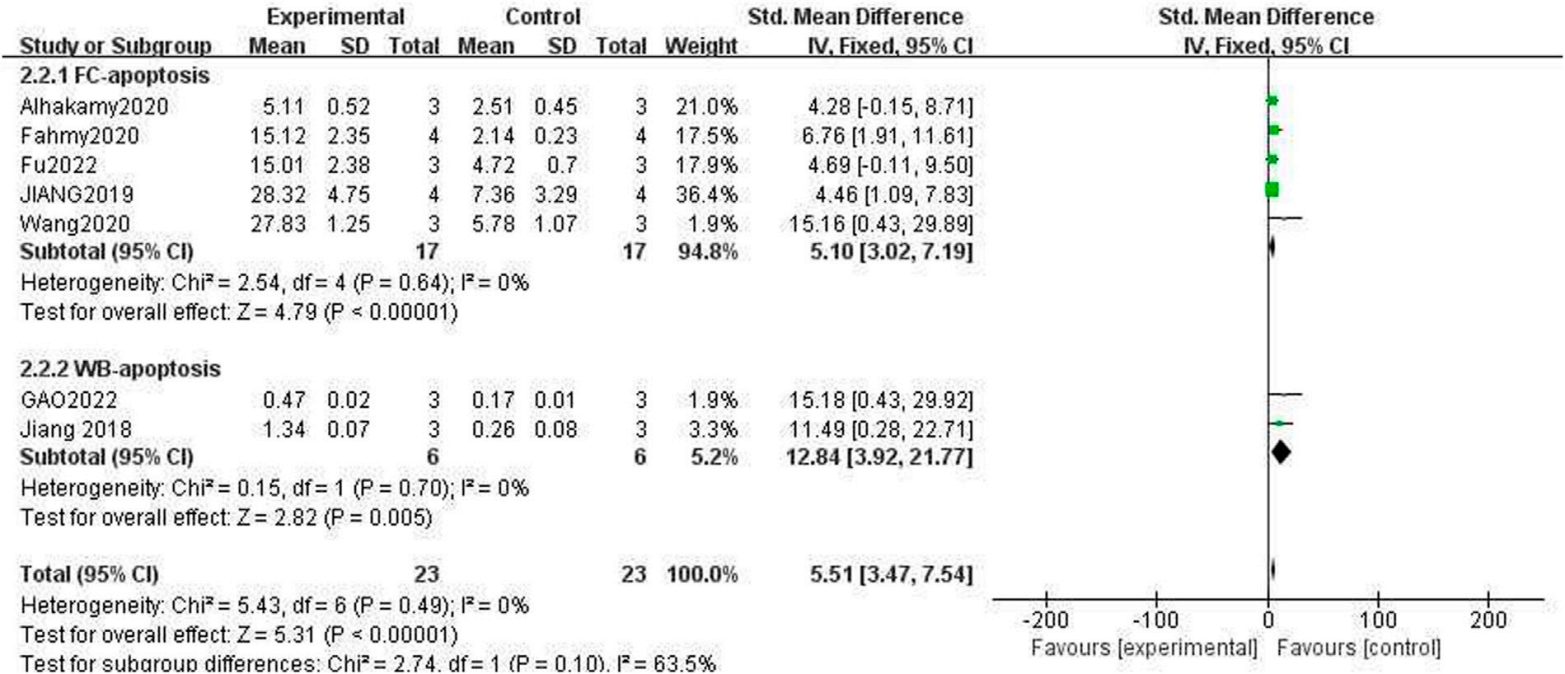
Pooled estimate of apoptosis induction ability in OC cells with ICA.

**FIGURE 8:**
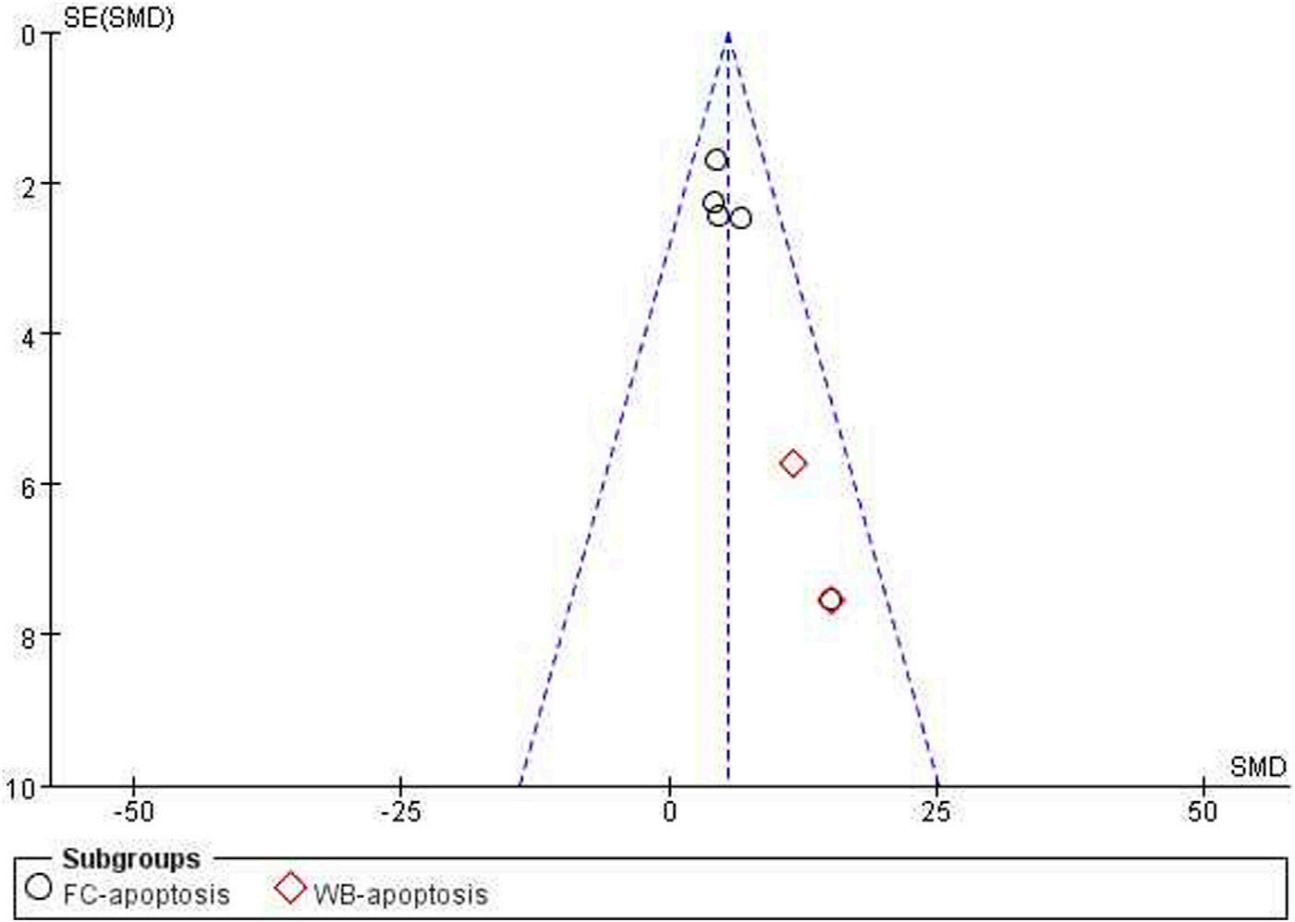
Funnel plot for the effects of ICA on OC cells in IC50.

**FIGURE 9:**
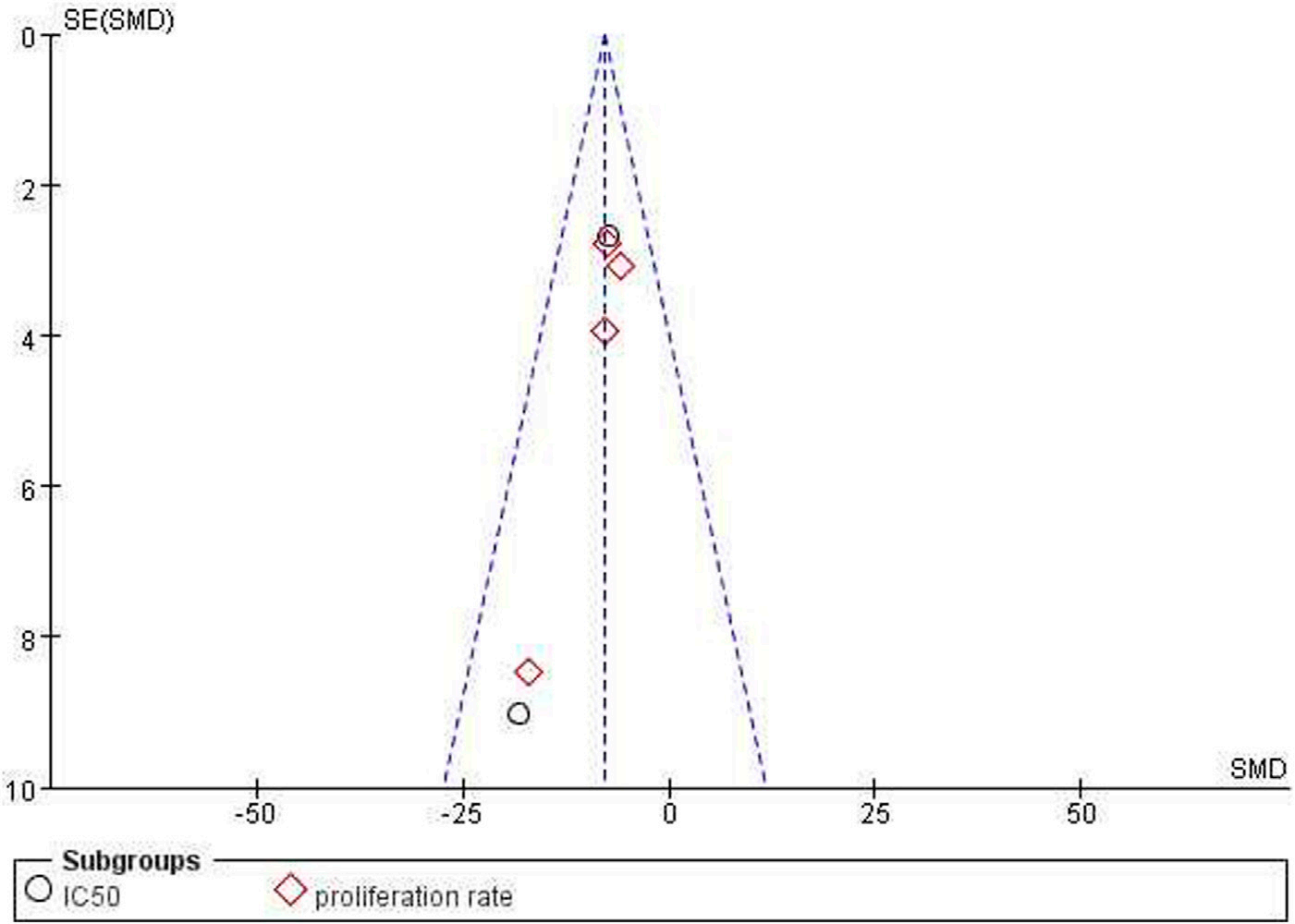
Funnel plot for the effects of ICA on OC cells in FC-apoptosis.

The test for subgroup differences yielded a result of *P = 0.10 > 0.05*, indicating that there was no significant difference between the groups and that the comparison between them was statistically significant. The combined results suggested that ICA could significantly enhance the induction of cancer cell apoptosis compared with the control group [*n = 23, SMD = 5.51, 95% CI (3.47, 7.54), p < 0.00001;* heterogeneity: *X* ^*2*^ *= 5.43, p = 0.49; I* ^*2*^ *= 0%*, Figure 7]. The funnel plots indicated asymmetry in the effects of ICA on apoptosis induction ability (Figures 8, 9). Begg’s test revealed statistical significance (*p = 0.003*), as did Egger’s test [*95% CI *(*1.35, 2.32*)*; p = 0.000*].

### 3.11 Sensitivity analysis

**FIGURE 10:**
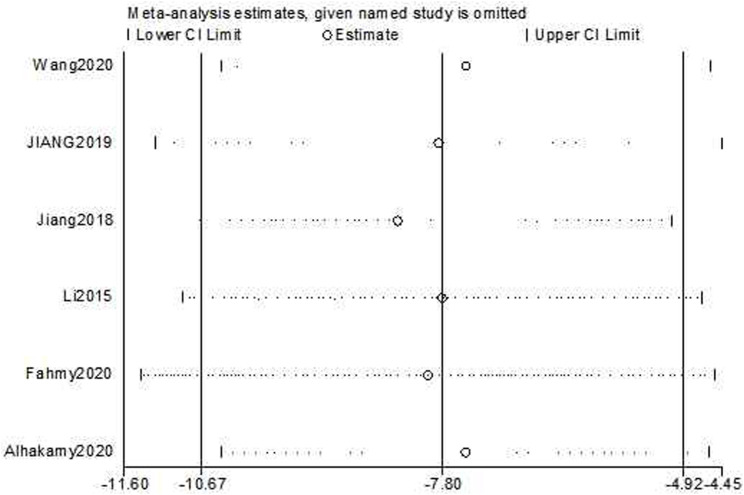
Sensitivity analysis plot for the effects of cell growth reduction on OC cells with ICA.

**FIGURE 11:**
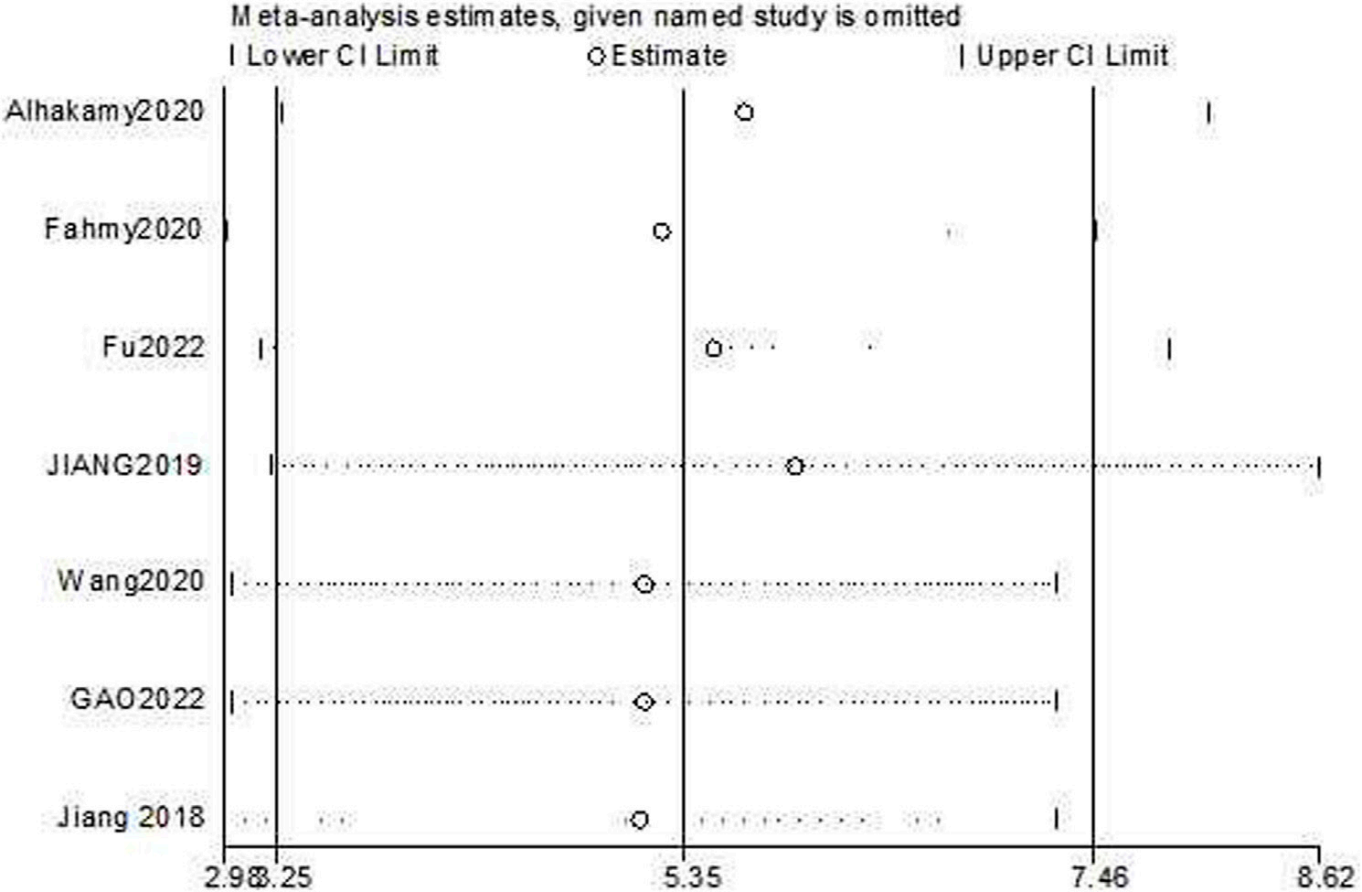
Sensitivity analysis plot for the effects of apoptosis induction on OC cells with ICA.

The sensitivity analyses of cell growth reduction and apoptosis induction ability were conducted by removing one study at each stage. The results indicated that no individual study significantly affected the pooled effect sizes. The sensitivity analysis of cell growth reduction ability results [*Estimate = −7.80, 95% CI *(*−10.67, −4.92*), Figure 10] indicated that there was significantly greater cell growth reduction in the experimental group compared to the control group. The sensitivity analysis of apoptosis induction [*Estimate = 5.35, 95% CI *(*3.25, 7.46*), Figure 11] indicated that there was significantly greater apoptosis induction in the experimental group compared to the control group.

## 4 Discussion

Network pharmacology is a new field in drug research (Yu et al., 2024). Conducting a network pharmacology analysis before conducting experiments on traditional Chinese medicine or natural drugs can more accurately determine the pathways that are involved in the mechanism of drug treatment of diseases (Xiong et al., 2024). A considerable volume of literature has confirmed the efficacy of ICA, which involves multiple pathways (Liu et al., 2021). Therefore, the pathways or genes identified in the literature should originate from genes and pathways that have been analysed in network pharmacology, so there should be an intersection of genes between the two gene sets (Wang Y. X. et al., 2022). To verify this hypothesis, the gene set obtained from network pharmacology was compared with the genes identified via experiments. The hypothesis was confirmed to be valid and it was concluded that the mechanism by which ICA acted in OC treatment involved these intersection genes and pathways.

Immunotherapy using T lymphocytes is an attractive strategy for treating various malignant tumors. However, due to the side effects and off-target effects of T cell immunotherapy, it is necessary to find a safe switch mechanism based on engineered T cells (Zhao et al., 2021). Adoptive cell therapy (ACT) using genetically engineered T cells has demonstrated high sensitivity, but some serious adverse events have been observed in several clinical studies (Jin et al., 2019). Optimal T-cell receptor (TCR) affinity in engineered T cells is crucial, and thus, the affinity of the receptor can determine the safety/efficacy of T-cell therapies (Xiang et al., 2021). Affinity is a major obstacle to the clinical success of adoptive cell therapy (ACT) due to the existence of targeted tumor extrinsic toxicity. When using antigen-specific receptors, affinity should be sufficiently high for proper T-cell activation in terms of efficacy (Yang et al., 2021). Conversely, interactions with low-affinity T-cell receptors are adequate to stimulate T cells, yet necessitate a higher affinity to sustain their proliferation. During phase I/II clinical trials of Adoptive Cell Transfer, T cells engineered with low affinity demonstrated safer profiles, albeit with diminished anti-tumor responses. Thus, the optimal affinity level is a pivotal determinant for balancing the safety and efficacy of Adoptive Cell Transfer (Wang F. et al., 2022).

Recent studies have expanded our understanding of ICA’s effects beyond ovarian cancer, highlighting its broad-spectrum anti-tumor potential: ICA has been shown to inhibit the proliferation of breast cancer cells by inducing apoptosis and cell cycle arrest. It downregulates the expression of Bcl-2 and upregulates Bax and caspase-3, promoting apoptotic pathways (Cheng et al., 2019). In non-small cell lung cancer (NSCLC), ICA suppresses tumor growth by inhibiting the PI3K/Akt/mTOR pathway. This inhibition leads to reduced cell proliferation and enhanced apoptosis, demonstrating ICA’s potential as a therapeutic agent in lung cancer treatment (Zhu and Ren, 2022). ICA has demonstrated anti-proliferative effects on prostate cancer cells by modulating the androgen receptor signaling pathway. It inhibits cell growth and induces apoptosis through the downregulation of AR and PSA expressions (Chen et al., 2023). Studies have shown that ICA can inhibit the growth and metastasis of colorectal cancer cells by modulating the Wnt/β-catenin signaling pathway. This pathway is crucial for cell proliferation and migration, and its inhibition by ICA leads to significant tumor suppression (Zhang et al., 2019). ICA has been found to induce apoptosis in leukemia cells by activating the intrinsic apoptotic pathway. It increases the expression of pro-apoptotic proteins like Bax and decreases anti-apoptotic proteins such as Bcl-2, thereby promoting cell death (Liu et al., 2023).

These studies offer a more comprehensive context for the application of ICA in ovarian cancer, illustrating its versatile anti-tumor properties across various cancer types. The mechanisms of action in these cancers frequently involve the modulation of key signaling pathways, including PI3K/Akt, Wnt/β-catenin, and intrinsic apoptotic pathways, akin to those observed in ovarian cancer.

The mechanism of action of ICA in treating ovarian cancer primarily involves multiple key signaling pathways. ICA activates the PI3K-Akt signaling pathway, which promotes cell survival and proliferation, and also enhances the sensitivity of cells to external signals, thereby inhibiting the spontaneous apoptosis of cancer cells to a certain extent. The regulation of the TNF signaling pathway by ICA is equally significant. It can increase the sensitivity of cells to apoptotic signals by affecting key proteins in this pathway, such as NF-κB. This dual regulatory effect allows ICA to demonstrate potent anti-tumor potential in the treatment of ovarian cancer. Through these mechanisms, ICA can not only inhibit the proliferation of ovarian cancer cells but also effectively promote programmed cell death, showcasing its potential as an adjunctive therapy. In contrast, the side effects of ICA are relatively mild, mainly manifested as mild gastrointestinal reactions and temporary blood pressure reduction, which makes ICA more acceptable to patients during long-term treatment. The anti-inflammatory and antioxidant properties of ICA provide additional protective effects in the treatment of ovarian cancer, helping to alleviate other complications caused by inflammation or oxidative stress. Therefore, ICA not only has potential in anti-tumor efficacy but its safety and pleiotropic effects also make it particularly important in modern ovarian cancer treatment.

The meta-analysis performed in this study aimed to ascertain the therapeutic efficacy of ICA on OC cells, revealing that treatment with ICA was significantly correlated with a reduction in cell growth [*SMD = −7.80, 95% CI *(*−10.67, −4.92*)] and apoptosis induction [*SMD = 5.51, 95% CI *(*3.47, 7.54*)]. The 14 overlapping genes identified from the literature and predicted by network pharmacology to be involved in the mechanisms through which ICA affects OC were primarily associated with the following pathways: pathways in cancer, PI3K-Akt signaling pathway, EGFR tyrosine kinase inhibitor resistance, hepatitis B, fluid shear stress and atherosclerosis, human papillomavirus infection, endocrine resistance, C-type lectin receptor signaling pathway, insulin resistance, HIF-1 signaling pathway, human T-cell leukemia virus 1 infection, TNF signaling pathway, measles, apoptosis, cellular senescence, and the sphingolipid signaling pathway. After excluding the 14 overlapping genes, the pathways corresponding to the remaining genes related to ICA’s impact on OC were also examined and encompassed: pathways in cancer, PI3K-Akt signaling pathway, endocrine resistance, hepatitis B, EGFR tyrosine kinase inhibitor resistance, melanoma, hepatocellular carcinoma, lipid and atherosclerosis, human cytomegalovirus infection, Kaposi sarcoma-associated herpesvirus infection, AGE-RAGE signaling pathway in diabetic complications, chronic myeloid leukemia, glioma, hepatitis C, cellular senescence, as well as other pathways pertinent to cell apoptosis and anti-tumor effects. This study offers a direction for future research in this area.

This study was subject to several limitations. Firstly, the meta-analysis encompassed only *in vitro* experiments, and due to the absence of a robust quality assessment framework for cellular studies, the conclusions drawn are inherently constrained. Secondly, the sensitivity analysis and asymmetry observed in the funnel plots indicated the presence of publication bias, which might have inflated the reported therapeutic effects of ICA. Thirdly, there is a paucity of research on the 187 intersection genes, which could introduce bias into the current findings. Incorporating additional data in the future may enhance our comprehension of this subject.

## 5 Conclusion

The study revealed that ICA exhibited a specific impact on anti-tumor function outcomes in comparison to placebo, characterized by enhanced cell growth reduction and the induction of apoptosis. Consequently, the pathways implicated in the therapeutic effects may be associated with cellular apoptosis and anti-tumor efficacy.

## Data Availability

The original contributions presented in the study are included in the article/Supplementary Material, further inquiries can be directed to the corresponding author.
