# Supplementary material for: Effect of icariin on ovarian cancer: a combined network pharmacology and meta-analysis of in vitro studies approach
Source: Front Pharmacol. 2024 Dec 20;15:1418111. doi: 10.3389/fphar.2024.1418111 (PMC11695863; doi:10.3389/fphar.2024.1418111)
Supplement: Supplementary file 1 [file DataSheet1.docx]

Supplementary Table 1. Relevant pathways and molecules involved in the inclusion of literature

|  | Atuor | Year | Related pathways and molecules involved |
| --- | --- | --- | --- |
| 1 | Wang | 2020 | TNF, MMP9, STAT3, PIK3CA, ERBB2, MTOR, IL2,PTGS2, KDR, F2 |
| 2 | Fu | 2022 | miR-1-3p, TNKS2, Wnt, b-catenin, cyclinD1, Survivin |
| 3 | JIANG | 2019 | LC3B, Beclin-1, ATG5, p62 |
| 4 | Jiang | 2018 | Beclin-1, LC3I/LC3II, ATG3, AMBRA1, mTOR |
| 5 | Li | 2015 | PTEN, RECK, Bcl-2, miR-21 |
| 6 | WANG | 2019 | FBP1, Cyclin E, p21, c-Myc, MMP9, CDK2, Cyclin A, WNT |
| 7 | Fahmy | 2020 | TNF-a |
| 8 | Alhakamy | 2020 |  |
| 9 | GAO | 2022 | AKT, NF-κB, p65 |

Supplementary Table 2. Molecules and corresponding genes included in the literature

| Molecules included in the literature | Corresponding genes to the Molecules |
| --- | --- |
| p62, ATG5, LC3B, Survivin, TNKS2, Wnt, b-catenin, p65, CyclinE, p21, c-Myc, MMP9, CDK2, STAT3, PIK3CA, ERBB2, MTOR, IL2, KDR, PTGS2, F2, Beclin-1, ATG3, AMBRA1, AKT, TNF-a, FBP1, PTEN, RECK, Bcl-2, NF-κB | GTF2H1, ATG5, MAP1LC3B, BIRC5, TNKS2, RSPO4, MCC, SYT1, CDKN1B, TCEAL1, MYC, MMP9, CDK2, STAT3, PIK3CA, ERBB2, MTOR, IL2, KDR, PTGS2, F2, BECN1, ATG3, AMBRA1, AKT1, TNF, FBP1, PTEN, RECK, BCL2, NFKB1, NFKB2, RELA, REL, RELB |

Supplementary Table 3. The top 20 key pathways corresponding to ICA and OC intersection genes which have not been verified yet

|  | Term | Count | P-Value | Genes |
| --- | --- | --- | --- | --- |
| 1 | hsa05200:Pathways in cancer | 77 | 1.36E-48 | RB1, ALK, GSK3B, FLT3, FLT4, PIK3CB, IGF1R, CASP9, RASSF1, CASP7, EDNRA, CASP8, CCND1, CASP3, EP300, JAK2, PRKACA, JAK1, PDGFRA, MAP2K1, HSP90AA1, DAPK1, MMP1, MMP2, F2R, PRKCA, FOS, RHOA, TGFBR1, TGFBR2, CCNA2, AR, IFNG, CCNE1, KIT, PPARG, RAF1, MET, TP53, GSTP1, XIAP, PIK3R1, HIF1A, EGFR, MAPK8, TERT, ABL1, E2F1, CTNNA1, E2F2, MAPK1, MAPK3, NQO1, GSTM2, GSTM1, TGFB1, NOS2, EGF, STAT1, IGF2, BRAF, IGF1, ESR1, ESR2, PTK2, VEGFA, NFKBIA, IL4, IL6, CDK6, CDK4, MDM2, BAX, FGFR2, NFE2L2, FGFR1, BCL2L1 |
| 2 | hsa05215:Prostate cancer | 31 | 8.72E-29 | RB1, GSK3B, GSTP1, PLAT, PIK3CB, PIK3R1, EGFR, IGF1R, CASP9, CCND1, PLAU, E2F1, E2F2, MAPK1, EP300, MAPK3, PDGFRA, MAP2K1, HSP90AA1, EGF, MMP3, BRAF, IGF1, NFKBIA, AR, CCNE1, MDM2, RAF1, TP53, FGFR2, FGFR1 |
| 3 | hsa05212:Pancreatic cancer | 26 | 7.65E-25 | RB1, PIK3CB, PIK3R1, EGFR, CASP9, MAPK8, CCND1, E2F1, E2F2, MAPK1, JAK1, MAPK3, MAP2K1, TGFB1, EGF, STAT1, BRAF, TGFBR1, TGFBR2, VEGFA, CDK6, CDK4, BAX, RAF1, TP53, BCL2L1 |
| 4 | hsa05219:Bladder cancer | 21 | 3.18E-24 | RB1, MAP2K1, DAPK1, MMP1, SRC, EGF, MMP2, BRAF, EGFR, TYMP, VEGFA, RASSF1, CCND1, CDK4, MDM2, E2F1, E2F2, MAPK1, RAF1, TP53, MAPK3 |
| 5 | hsa01522:Endocrine resistance | 27 | 4.36E-23 | RB1, SRC, PIK3CB, PIK3R1, EGFR, IGF1R, MAPK8, CCND1, E2F1, E2F2, MAPK1, PRKACA, MAPK3, MAP2K1, MMP2, BRAF, IGF1, FOS, MAPK14, ESR1, PTK2, ESR2, CDK4, MDM2, BAX, RAF1, TP53 |
| 6 | hsa05161:Hepatitis B | 32 | 8.86E-23 | RB1, PCNA, SRC, PIK3CB, PIK3R1, CASP9, MAPK8, CASP8, CASP3, E2F1, E2F2, MAPK1, EP300, JAK2, JAK1, MAPK3, MAP2K1, TGFB1, STAT1, PRKCA, BRAF, FOS, MAPK14, TGFBR1, TGFBR2, NFKBIA, CCNA2, IL6, CCNE1, BAX, RAF1, TP53 |
| 7 | hsa04151:PI3K-Akt signaling pathway | 43 | 2.52E-22 | GSK3B, FLT1, FLT3, FLT4, PIK3CB, PIK3R1, EGFR, PIK3CG, IGF1R, CASP9, ERBB3, CCND1, SPP1, MAPK1, JAK2, JAK1, MAPK3, PDGFRA, MAP2K1, HSP90AA1, EGF, NOS3, INSR, F2R, IGF2, PRKCA, IGF1, PTK2, VEGFA, IL4, IL6, CDK6, CCNE1, CDK4, KIT, MDM2, RAF1, MET, TP53, FGFR2, FGFR1, BCL2L1, EPHA2 |
| 8 | hsa01521:EGFR tyrosine kinase inhibitor resistance | 24 | 1.56E-21 | PDGFRA, GSK3B, MAP2K1, SRC, EGF, BRAF, PRKCA, PIK3CB, PIK3R1, IGF1, EGFR, IGF1R, VEGFA, IL6, ERBB3, BAX, MAPK1, JAK2, RAF1, MET, FGFR2, JAK1, BCL2L1, MAPK3 |
| 9 | hsa05218:Melanoma | 23 | 3.75E-21 | RB1, PDGFRA, MAP2K1, EGF, BRAF, PIK3CB, PIK3R1, IGF1, EGFR, IGF1R, CDK6, CCND1, CDK4, MDM2, E2F1, BAX, E2F2, MAPK1, RAF1, MET, TP53, FGFR1, MAPK3 |
| 10 | hsa05225:Hepatocellular carcinoma | 31 | 3.82E-21 | RB1, GSK3B, GSTP1, PIK3CB, PIK3R1, EGFR, IGF1R, CCND1, TERT, E2F1, E2F2, MAPK1, MAPK3, NQO1, GSTM2, MAP2K1, GSTM1, TGFB1, IGF2, PRKCA, BRAF, TGFBR1, TGFBR2, CDK6, CDK4, BAX, RAF1, MET, TP53, BCL2L1, NFE2L2 |
| 11 | hsa05417:Lipid and atherosclerosis | 34 | 4.57E-21 | GSK3B, SRC, PIK3CB, PIK3R1, ICAM1, CASP9, CASP7, MAPK8, CASP8, CASP3, CCL5, MAPK1, JAK2, MAPK3, HSP90AA1, MMP1, NOS3, MMP3, PRKCA, FOS, MAPK14, SOD2, RHOA, PTK2, ERN1, NFKBIA, IL6, IL1B, CYP1A1, BAX, PPARG, TP53, BCL2L1, NFE2L2 |
| 12 | hsa05163:Human cytomegalovirus infection | 34 | 1.97E-20 | RB1, GSK3B, SRC, PIK3CB, PIK3R1, EGFR, CASP9, CASP8, CCND1, CASP3, CCL5, CXCR2, E2F1, E2F2, MAPK1, PRKACA, JAK1, MAPK3, PDGFRA, MAP2K1, PRKCA, MAPK14, RHOA, PTK2, VEGFA, NFKBIA, IL6, CDK6, CDK4, IL1B, MDM2, BAX, RAF1, TP53 |
| 13 | hsa05167:Kaposi sarcoma-associated herpesvirus infection | 32 | 2.41E-20 | RB1, GSK3B, SRC, PIK3CB, PIK3R1, HIF1A, PIK3CG, ICAM1, CASP9, MAPK8, CASP8, CCND1, CASP3, E2F1, E2F2, MAPK1, EP300, JAK2, JAK1, MAPK3, MAP2K1, STAT1, FOS, MAPK14, VEGFA, NFKBIA, IL6, CDK6, CDK4, BAX, RAF1, TP53 |
| 14 | hsa04933:AGE-RAGE signaling pathway in diabetic complications | 25 | 3.06E-20 | SERPINE1, PIK3CB, PIK3R1, ICAM1, MAPK8, CCND1, CASP3, MAPK1, JAK2, MAPK3, TGFB1, NOS3, STAT1, MMP2, PRKCA, MAPK14, F3, TGFBR1, TGFBR2, VEGFA, IL1A, IL6, CDK4, IL1B, BAX |
| 15 | hsa05223:Non-small cell lung cancer | 22 | 9.33E-20 | RB1, ALK, MAP2K1, EGF, BRAF, PRKCA, PIK3CB, PIK3R1, EGFR, CASP9, RASSF1, CDK6, CCND1, CDK4, E2F1, BAX, E2F2, MAPK1, RAF1, MET, TP53, MAPK3 |
| 16 | hsa05205:Proteoglycans in cancer | 32 | 1.29E-19 | SRC, PIK3CB, PIK3R1, HIF1A, EGFR, IGF1R, ERBB3, CCND1, PLAU, CASP3, MAPK1, PRKACA, MAPK3, MAP2K1, TGFB1, CAV1, MMP2, IGF2, PRKCA, BRAF, IGF1, MAPK14, ESR1, RHOA, PTK2, VEGFA, MDM2, PDCD4, RAF1, MET, TP53, FGFR1 |
| 17 | hsa05214:Glioma | 22 | 2.41E-19 | RB1, PDGFRA, MAP2K1, EGF, BRAF, PRKCA, PIK3CB, PIK3R1, IGF1, EGFR, IGF1R, CDK6, CCND1, CDK4, MDM2, E2F1, BAX, E2F2, MAPK1, RAF1, TP53, MAPK3 |
| 18 | hsa05220:Chronic myeloid leukemia | 22 | 3.28E-19 | RB1, MAP2K1, TGFB1, BRAF, PIK3CB, PIK3R1, TGFBR1, TGFBR2, NFKBIA, CDK6, CCND1, CDK4, MDM2, E2F1, ABL1, BAX, E2F2, MAPK1, RAF1, TP53, BCL2L1, MAPK3 |
| 19 | hsa04218:Cellular senescence | 28 | 1.01E-18 | RB1, SERPINE1, PIK3CB, PIK3R1, CCNB1, CCND1, CHEK2, CHEK1, E2F1, E2F2, MAPK1, MAPK3, MAP2K1, TGFB1, IGFBP3, MAPK14, TGFBR1, TGFBR2, CCNA2, IL1A, IL6, CDK6, CCNE1, CDK4, CDK1, MDM2, RAF1, TP53 |
| 20 | hsa05160:Hepatitis C | 28 | 1.20E-18 | RB1, GSK3B, PIK3CB, PIK3R1, EGFR, CASP9, CASP8, CCND1, CASP3, E2F1, E2F2, MAPK1, JAK1, MAPK3, MAP2K1, EGF, STAT1, BRAF, NFKBIA, CXCL10, CLDN4, CDK6, IFNG, CDK4, BAX, RAF1, PPARA, TP53 |


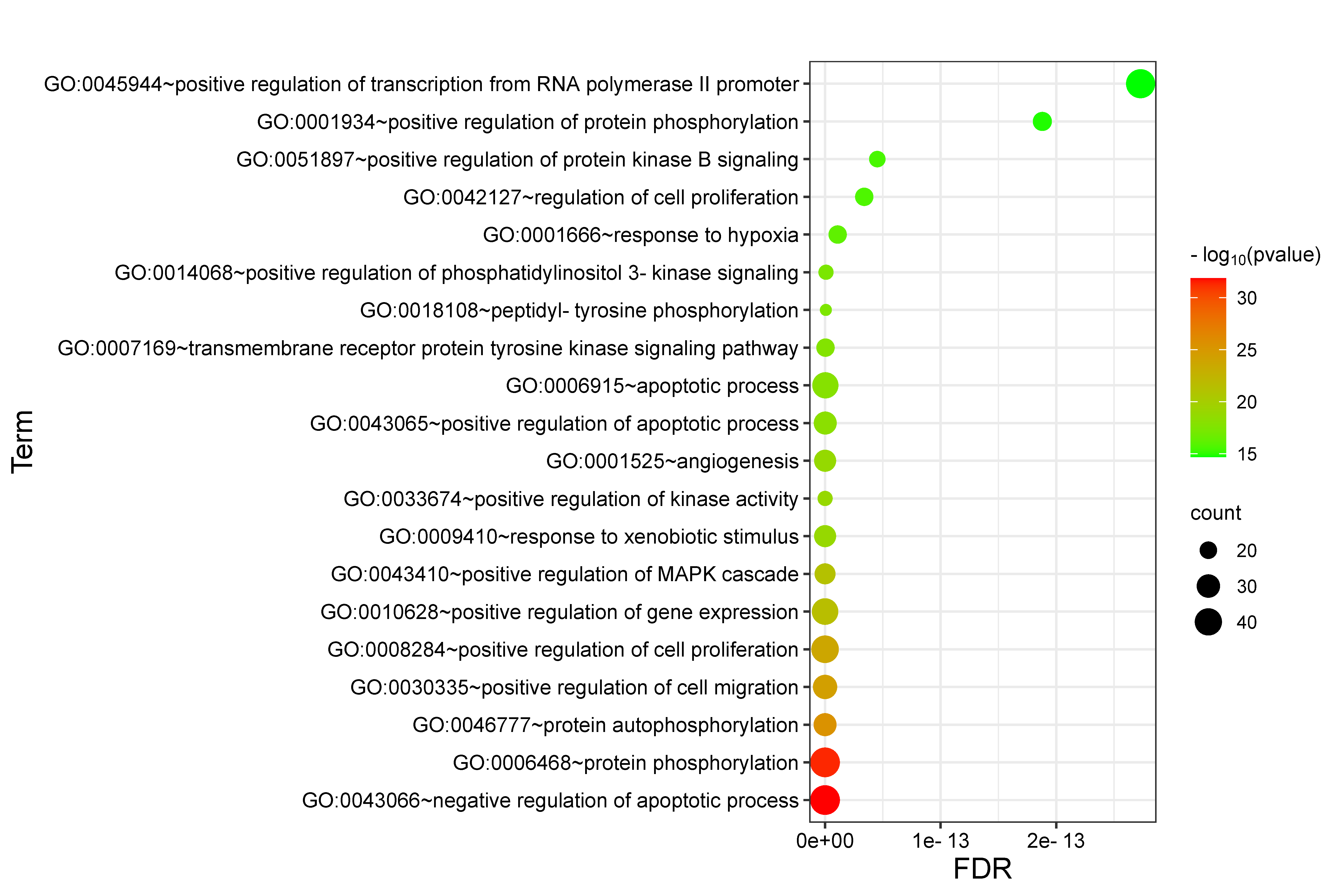


Figure S1. GO gene enrichment bubble chart. The top 20 of biological processes (BP) category for ICA and OC intersection genes.


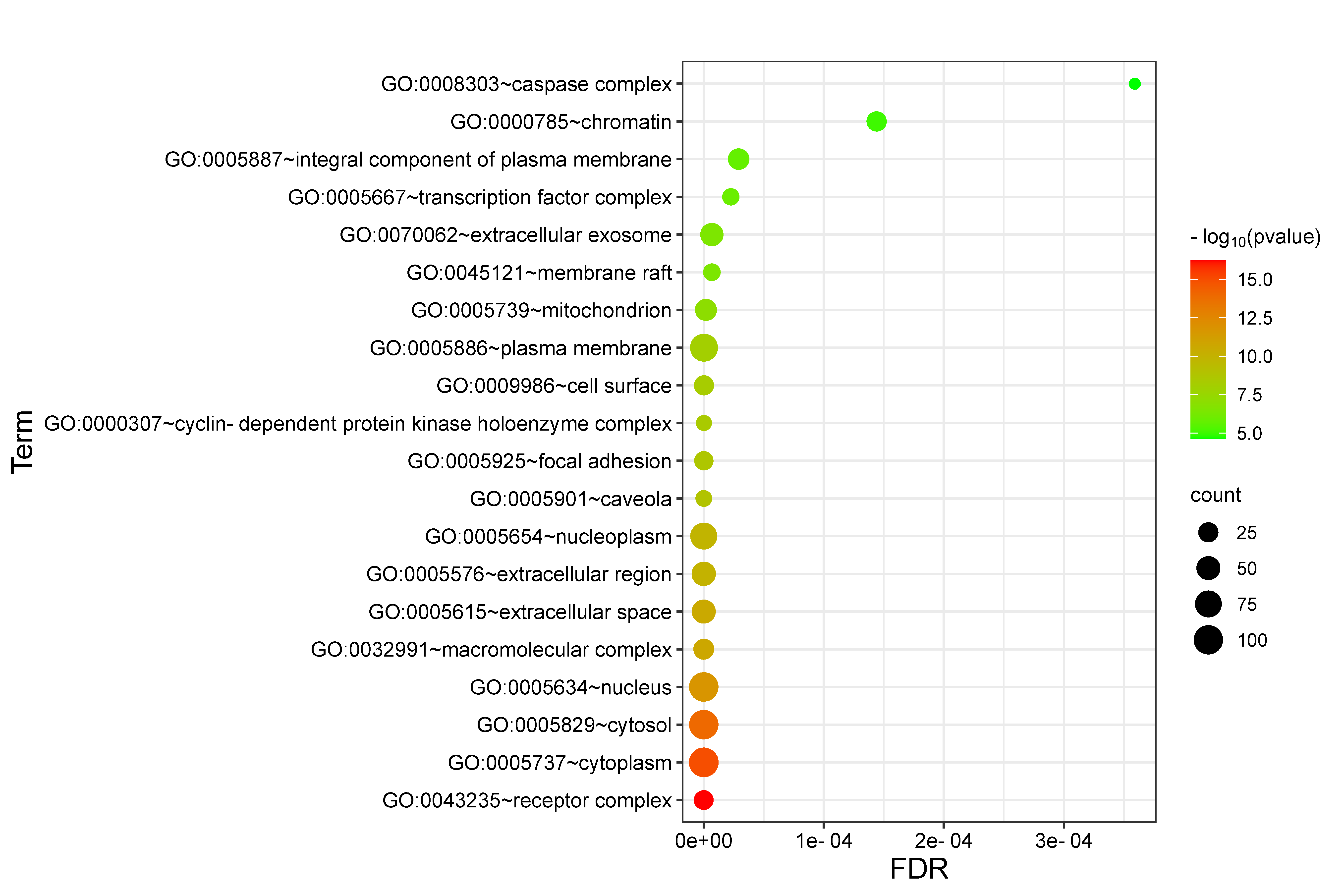


Figure S2. GO gene enrichment bubble chart. The top 20 of cellular ingredients (CC) category for ICA and OC intersection genes.


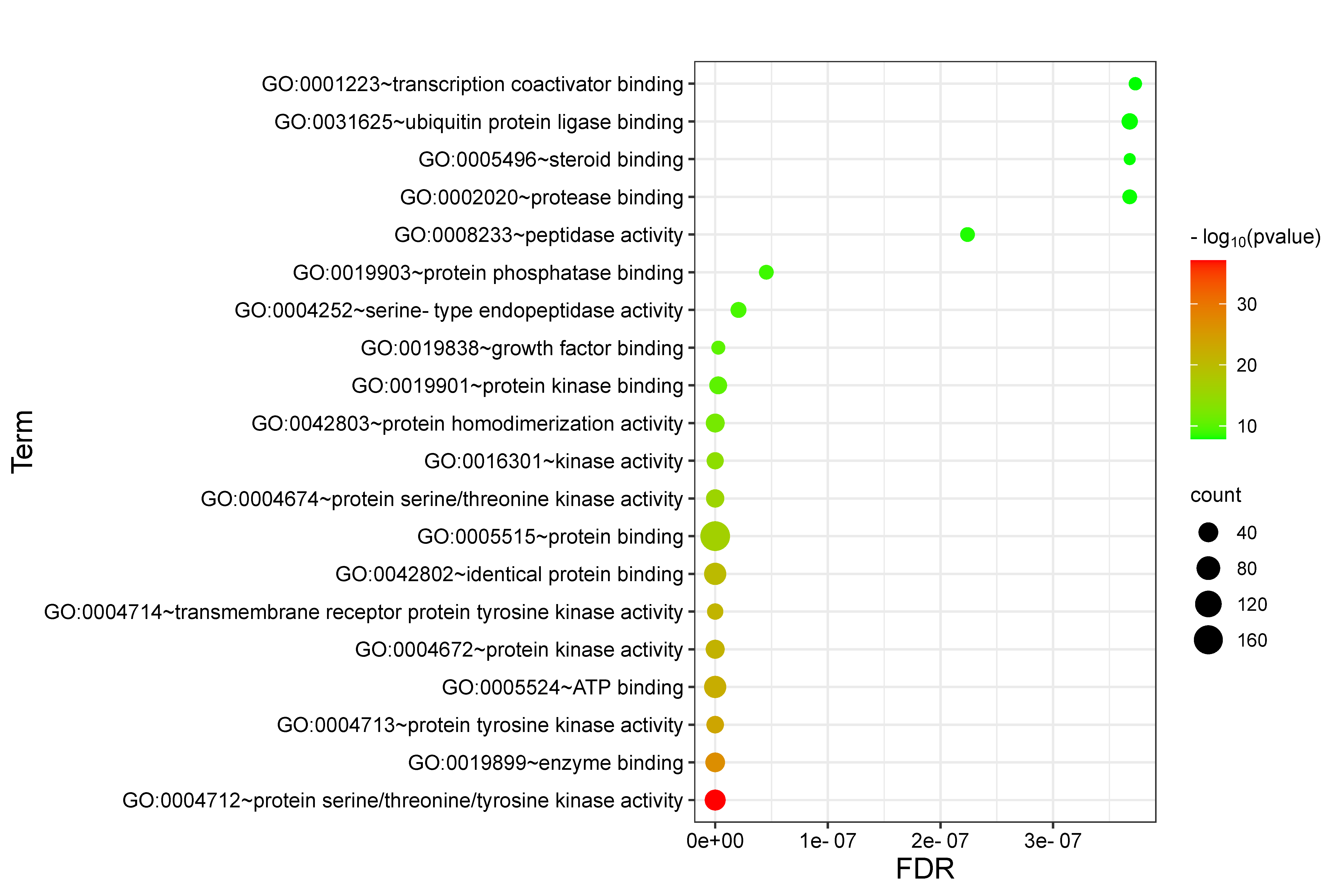


Figure S3. GO gene enrichment bubble chart. The top 20 of molecular functions (MF) category for ICA and OC intersection genes.


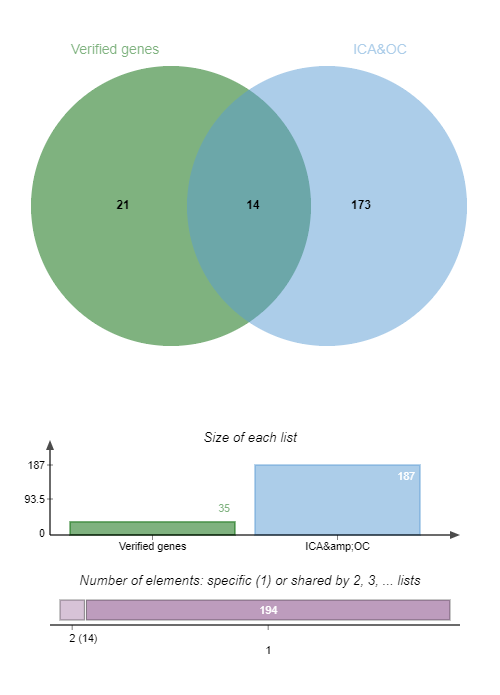


Figure S4. The intersection of genes involved in the included literature and predicted by network pharmacology genes which ICA acting on OC.


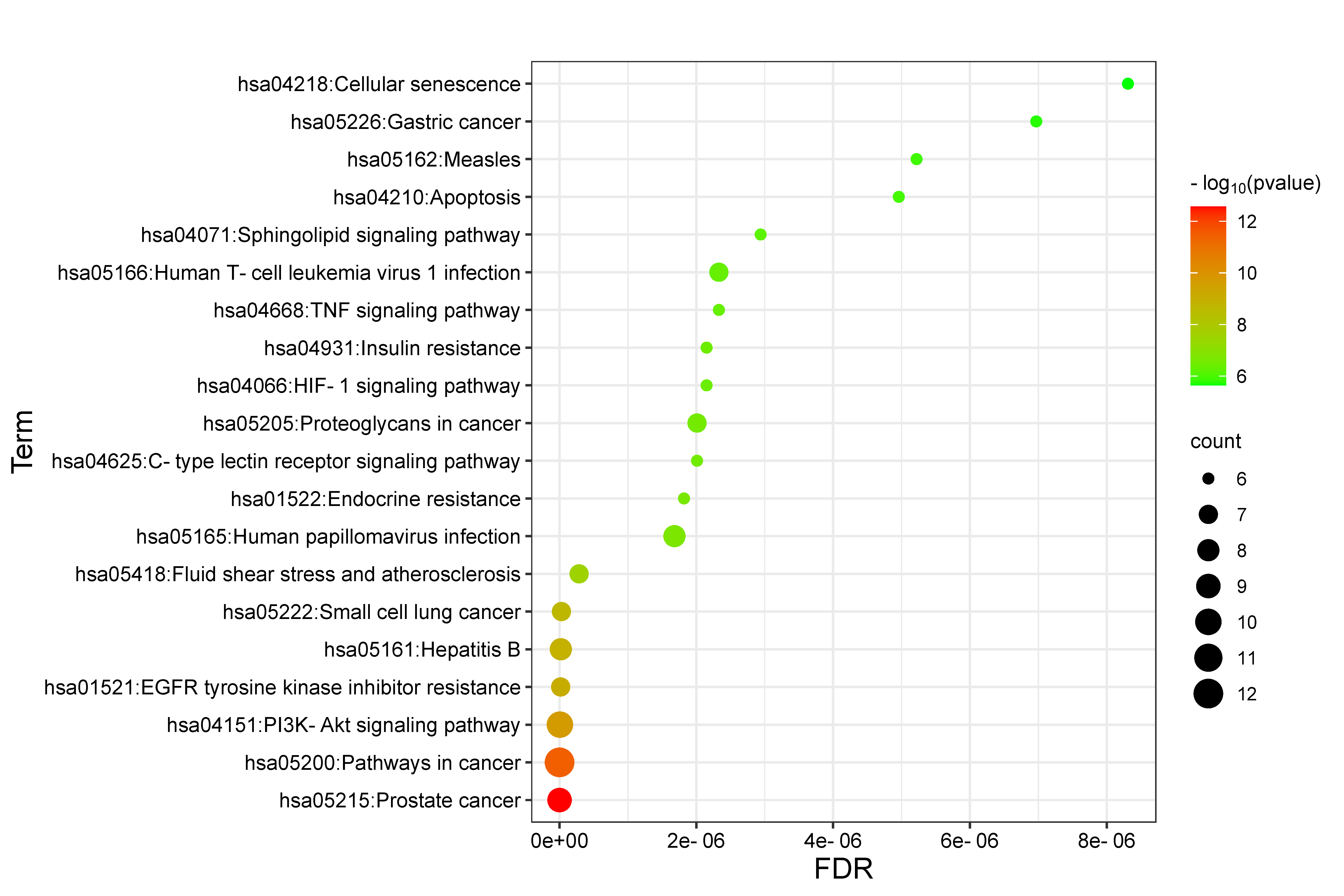


Figure S5. GO gene enrichment bubble chart. The top 20 key pathways corresponding to ICA and OC intersection genes which have been verified in the included literature.


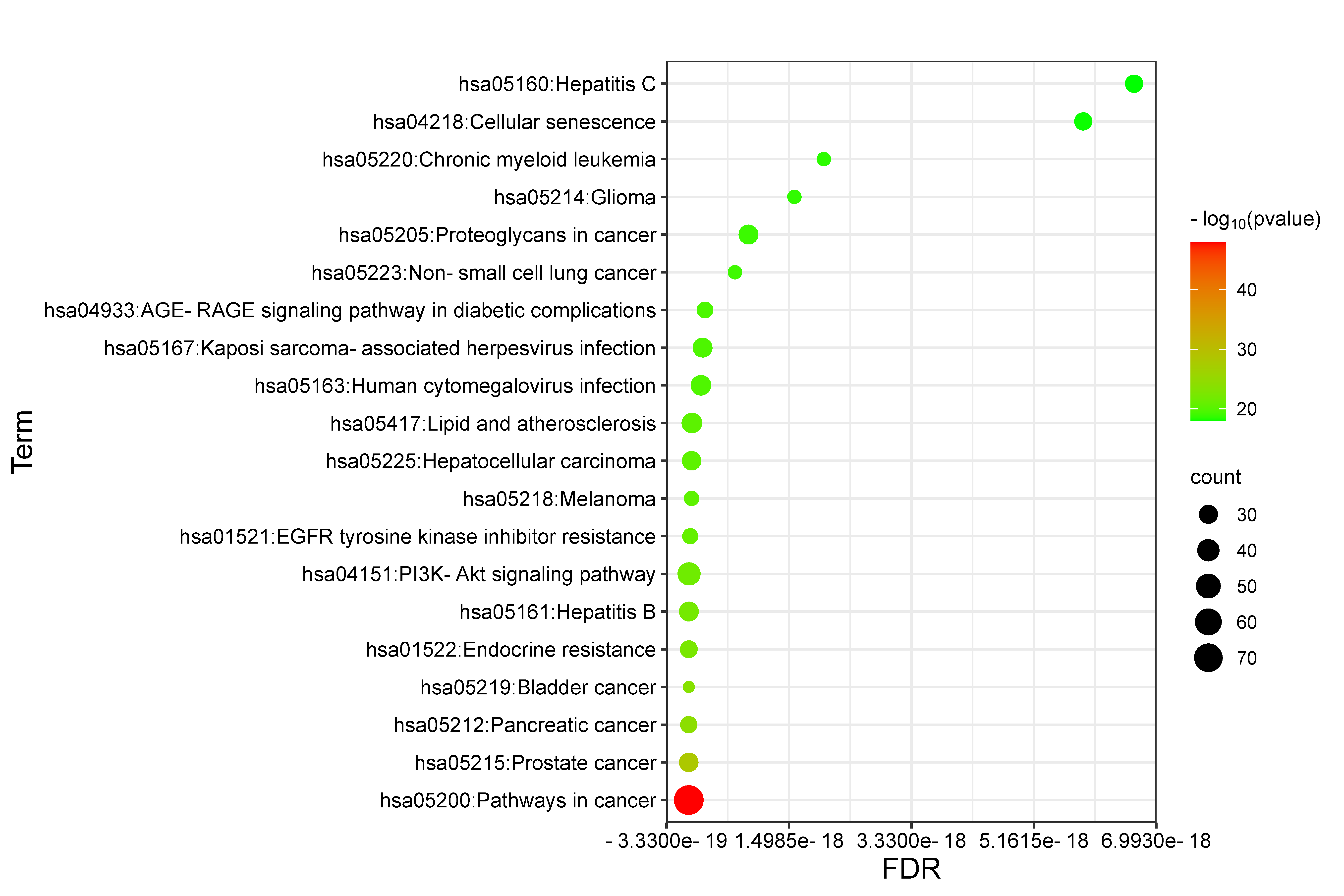


Figure S6. GO gene enrichment bubble chart. The top 20 key pathways corresponding to ICA and OC intersection genes which have not been verified yet.
